# Endometriosis-associated clear cell carcinoma arising in caesarean section scar: a case report and review of the literature

**DOI:** 10.1186/s12957-016-1054-7

**Published:** 2016-12-03

**Authors:** Gabriella Ferrandina, Eleonora Palluzzi, Francesco Fanfani, Stefano Gentileschi, Anna Lia Valentini, Maria Vittoria Mattoli, Ilaria Pennacchia, Giovanni Scambia, Gianfranco Zannoni

**Affiliations:** 1 Department Medicine and Health Sciences, University of Molise, Campobasso/Gynecologic Oncology Unit, Campobasso, Italy; 2 Gynecologic Oncology Unit, Fondazione “Policlinico Universitario A. Gemelli”, Rome, Italy; 3 Department Medicine and Aging Sciences, University “G D’Annunzio”, Chieti-Pescara, Italy; 4 Department Plastic and Reconstructive Surgery, Fondazione “Policlinico Universitario A. Gemelli”, Rome, Italy; 5 Department Radiological Sciences, Institute of Radiology, Catholic University, Rome, Italy; 6 Institute of Nuclear Medicine, Fondazione “Policlinico Universitario A. Gemelli”, Rome, Italy; 7 Department Pathology, Catholic University, Rome, Italy

## Abstract

**Background:**

Malignant transformation has been reported in approximately 1% of the endometriosis cases; herein, we report a case of clear cell endometrial carcinoma arising from endometriosis foci located within a caesarean section scar.

**Case presentation:**

In November 2014, a Caucasian, 44-year-old woman was transferred to our institution because of severe respiratory failure due to massive lung embolism and rapid enlargement of a subcutaneous suprapubic mass. Abdomino-pelvic magnetic resonance showed a 10.5 × 5.0 × 5.0 cm subcutaneous solid mass involving the rectus abdominis muscle. Pelvic organs appeared normal, while right external iliac lymph nodes appeared enlarged (maximum diameter = 16 mm). A whole-body positron emission tomography/computed tomography scan showed irregular uptake of the radiotracer in the 22 cm mass of the abdominal wall, and in enlarged external iliac and inguinal lymph nodes. In December 2014, the patient underwent exploratory laparoscopy showing normal adnexae and pelvic organs; peritoneal as well as cervical, endometrial and vesical biopsies were negative. The patient was administered neo-adjuvant chemotherapy with carboplatin and paclitaxel, weekly, without benefit and then underwent wide resection of the abdominal mass, partial removal of rectus abdominis muscle and fascia, radical hysterectomy, bilateral salpingo-oophorectomy, and inguinal and pelvic lymphadenectomy. The muscular gap was repaired employing a gore-tex mesh while the external covering was made by a pedicled perforator fasciocutaneous anterolateral thigh flap. Final diagnosis was clear cell endometrial adenocarcinoma arising from endometriosis foci within the caesarean section scar. Pelvic and inguinal lymph nodes were metastatic. Tumor cells were positive for CK7 EMA, CKAE1/AE3, CD15, CA-125, while immunoreaction for Calretinin, WT1, estrogen, and progesterone receptors, cytokeratin 20, CD10, alpha fetoprotein, CDX2, TTF1, and thyroglobulin were all negative. Liver relapse occurred after 2 months; despite 3 cycles of pegylated liposomal doxorubicin (20 mg/m^2^, biweekly administration), the death of the patient disease occurred 1 month later.

**Conclusions:**

Attention should be focused on careful evaluation of patient history in terms of pelvic surgery, and symptoms suggestive of endometriosis such as repeated occurrence of endometriosis nodules at CS scar, or cyclic pain, or volume changes of the nodules.

## Background

Malignant transformation has been reported in approximately 1% of the endometriosis cases, and most frequently this transformation takes place at the ovary, accounting for about 80% of the endometriosis-associated malignancies [1].

Endometriosis occurring in surgical abdominal scar has been mainly documented after caesarean section (CS) or hysterectomy (0.03 up to 0.4%), and its malignant transformation is very rare [2]: clear cell histology accounts for only 4.5% of extragonadal endometriosis-associated malignancies, while representing the most common histotype in case of parietal localization [1].

Given the rarity of malignant transformation of abdominal scar endometriosis to clear cell histology, pathogenesis and risk factors of this disease are hardly assessable [3]. Nonetheless, due to the increased rate of CS registered in the last years, we can expect a parallel increase of endometriosis implants in the CS scar and occurrence of clear cell carcinoma (CCC) of the abdominal wall.

Herein, we report a new case of CCC arising from endometriosis foci located within a CS scar; a systematic review of the available literature relative to this issue is also presented.

## Case presentation

In November 2014, a Caucasian, 44-year-old woman was transferred to our institution from the emergency unit of another hospital where she had been successfully treated for a severe respiratory failure due to massive lung embolism and cardiogenic shock.

Her familial history was uneventful; she had undergone one caesarean section 9 years before without complications and had assumed oral contraceptives until the appearance of symptoms. She had never suffered from signs or symptoms of endometriosis.

The patient referred to have documented, since the last 5 months, the slow enlargement of a suprapubic mass at the CS scar (lower abdominal incision) and abdominal swelling.

In July 2014, she had already performed abdomino-pelvic magnetic resonance imaging (MRI) showing a 10.5×5.0×5.0 cm subcutaneous solid mass with cystic areas and internal septa involving the rectus abdominis muscle. The mass appeared strictly adherent to the uterus and recto-sigma. Pelvic organs appeared normal, while right external iliac lymph nodes appeared enlarged (short axis maximum diameter = 16 mm).

The patient had been already triaged to fine needle aspiration (FNA) of the mass which was suggestive of endometrial tubule-papillary carcinoma.

**Fig. 1:**
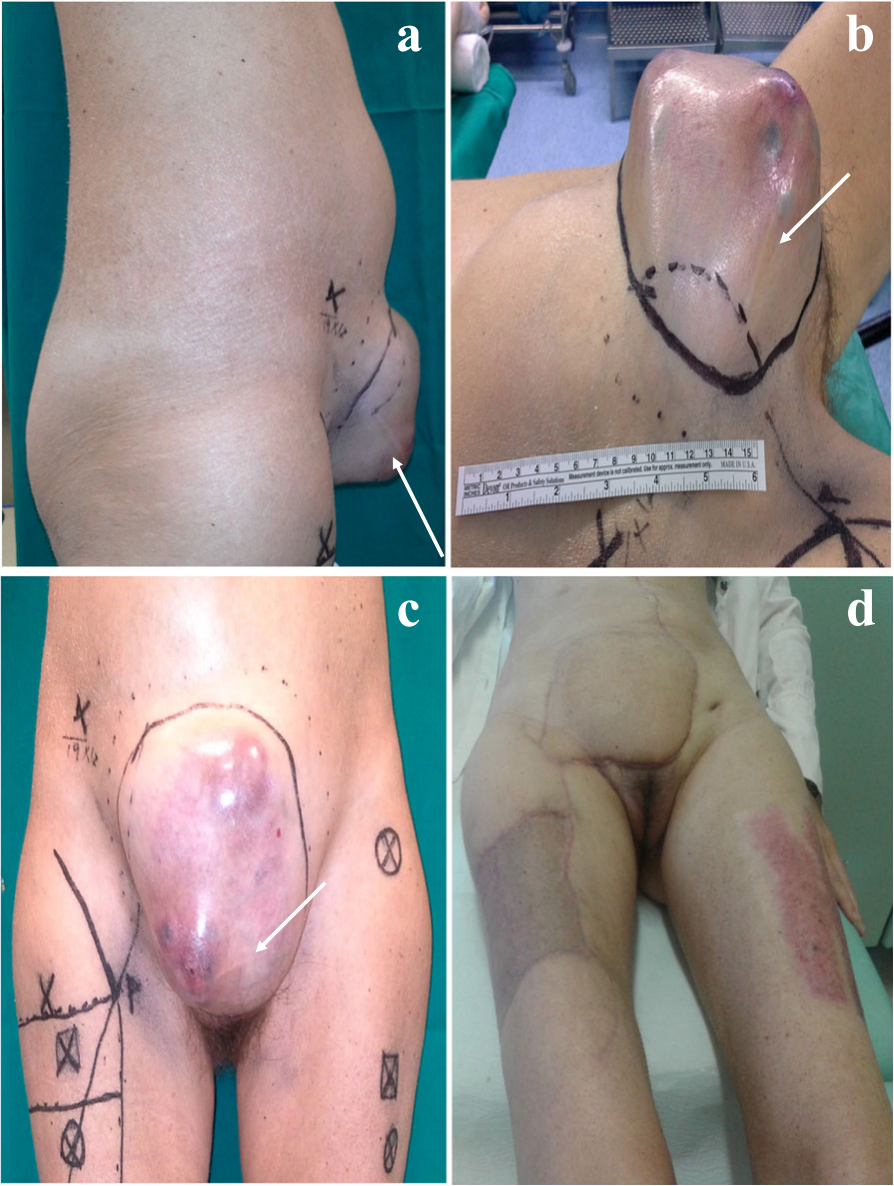
Clinical appearance of the patient at time of presentation (**a**–**c**): a large mass (**a**, **b**) extended from the pubic symphysis to the umbilicus was evident; the mass appeared mainly solid with some cystic lesions on the surface. The caesarean section scar is evident (*white arrow*). Appearance of the abdominal reconstruction after 2 months from surgery (**d**)

At physical examination, a suprapubic mass of almost 20 cm maximum diameter was documented close to the midpoint of the CS scar (Fig. 1a–c). Laboratory tests revealed normal levels of CEA, CA125, and squamous cell carcinoma antigen.

**Fig. 2:**
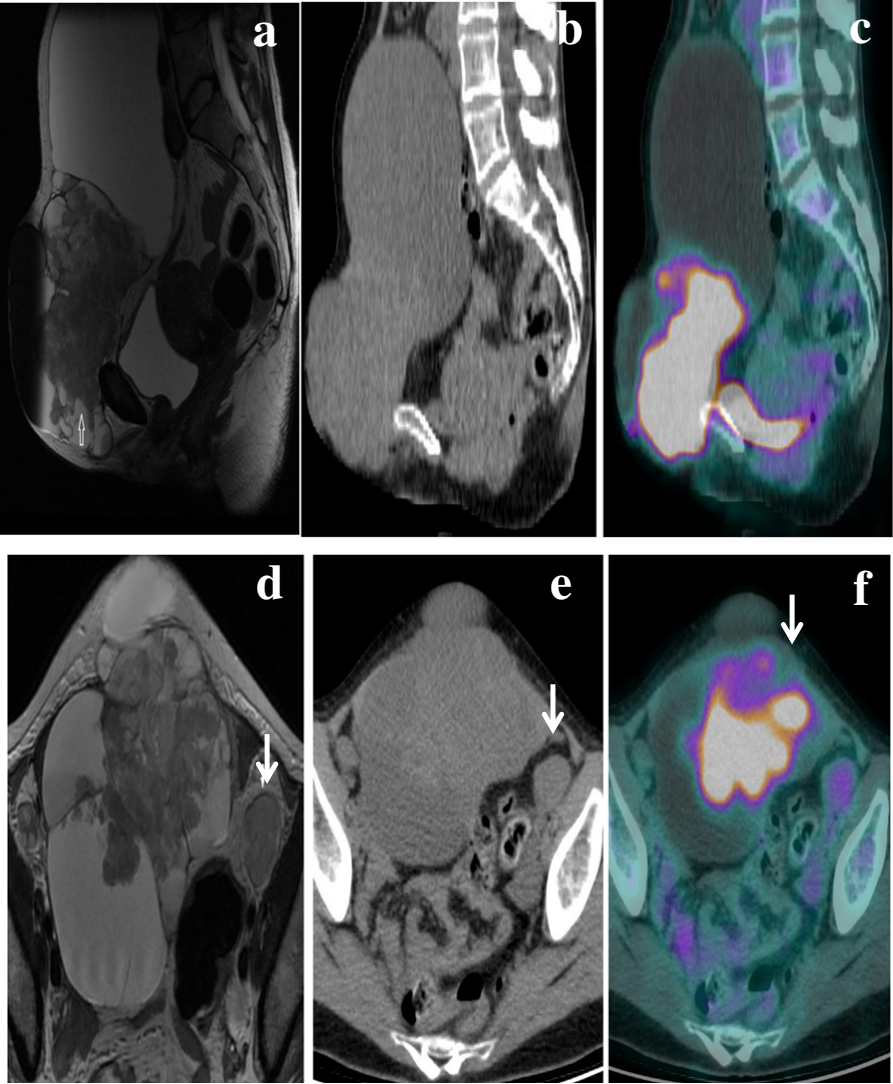
Pre-surgical T2-weighted FSE sagittal MRI (**a**) documenting a supra-vesical pelvic mass extending into the anterior pelvic wall and showing non-homogeneous signal intensity and solid components. Sagittal CT (**b**) and fused ^18^F-FDG PET/CT (**c**) images showed non-homogeneous uptake in the 22 cm abdominal mass: in particular, intense abnormal ^18^F-FDG uptake was present in the caudal solid component of the mass, whereas an absent area of tracer uptake was evident in its cranial fluid component. Corresponding T2-weighted FSE axial MRI (**d**) confirmed the presence of a solid and partially fluid pelvic mass, and enlarged loco-regional lymph nodes (*arrow*). Axial CT (**e**) and fused ^18^F-FDG PET/CT images (**f**) showed intense tracer uptake in the solid component of the mass, extended up to the skin surface of pelvis, and enlarged left external iliac lymph-node, characterized by increased tracer uptake (*arrows*)

Once the patient recovered from the acute phase of lung embolism and achieved haemodynamic compensation, a whole-body positron emission tomography/computed tomography (PET/CT) scan was performed about 60 min after the intravenous administration of ^18^F-FDG (148 MBq), showing irregular/non-homogeneous uptake of the radiotracer in the 22 cm mass of the abdominal wall. Increased ^18^F-FDG uptake was also seen in enlarged external iliac and inguinal lymph nodes bilaterally (Fig. 2b, c, e, f). Abdomino-pelvic MRI performed in our institution documented normal uterus and adnexae, and confirmed the presence of the anterior abdominal wall mass composed of solid as well as locular areas; the mass completely infiltrated the rectus muscle of abdomen and extended up to the skin surface, while displacing intestinal loops. The cleavage planes neighboring the uterus and bladder appeared preserved as well as the inguinal, external iliac, and obturator lymph nodes (Fig. 2a, d).

In December 2014, the patient underwent exploratory laparoscopy showing normal adnexae and pelvic organs; peritoneal as well as cervical, endometrial, and vesical biopsies were negative.

Considering the results of FNA suggesting primary Müllerian-derived carcinoma and the extent of disease whose radical resection would require widely demolitive surgical procedures, the patient was triaged to neo-adjuvant chemotherapy with carboplatin (AUC = 2), and paclitaxel (80 mg/m^2^), every week. After 3 cycles (9 administrations) MRI documented only a slight reduction of the mass and lymphadenopathies, thus leading to consider the need of a multidisciplinary approach with gynecologic and medical oncologists, surgeons, and plastic surgeons in order to plan the most adequate patient treatment. After a thorough evaluation, radical surgery with reconstruction was planned, and an extensive counseling was carried out with the patient and her relatives.

At the beginning of March 2015, the patient underwent wide resection of the abdominal mass, partial removal of rectus abdominis muscle and fascia; moreover, radical hysterectomy, bilateral salpingo-oophorectomy (BSO) as well as inguinal and pelvic lymphadenectomy were performed.

At the end of surgery, the abdominal wall defect appeared as a round hole of 14 × 14 cm which involved the abdominal wall thickness completely. The muscular gap was repaired employing a gore-tex mesh anchored directly on the residual rectus muscles and sheets. The external covering had been planned by a pedicled perforator fasciocutaneous anterolateral thigh flap. The perforator vessels from the descending branch of the lateral circumflex femoral artery had been preoperatively located, with ultrasound doppler sonography, at the level of the intermuscular lateral septum of the thigh. The right thigh was chosen as donor site because the perforators were more distal, thus allowing to lengthen the pedicle. The donor site, on the right thigh, was repaired both by direct closure, and a skin graft harvested from the contralateral thigh. Postoperative course of the wounds was uneventful and stitches were removed at 2 weeks. The patient was discharged after 18 days in good clinical conditions, and the cosmetic result was acceptable (Fig. 1d).

**Fig. 3:**
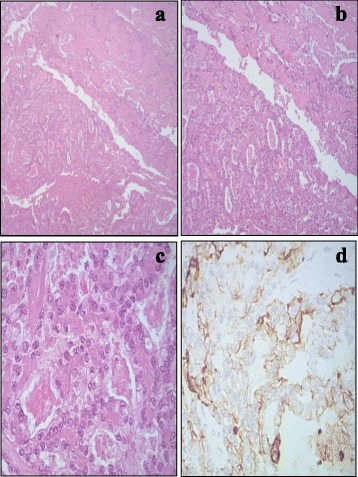
The tumor displayed a mixed pattern of both tubulocystic and papillary growth, HE x5 (**a**), and HEx10 (**b**); cells were large with abundant clear or occasionally eosinophilic cytoplasm. Note the high-grade nuclear atypia, HEx20 (**c**); neoplastic cells showed positive cell membrane immunostaining for CK7 (x20) (**d**)

Macroscopically, the surgical specimen consisted of cutis and subcutaneous tissue which contained a capsulated tumor mass measuring 18 cm in maximum diameter, infiltrating the rectus abdominis muscle. The cut surface was whitish and showed areas of necrosis and hemorrhage. Histological examination of tissue samples revealed neoplastic proliferation of large-sized, epitheliomorphous cells with abundant clear or occasionally eosinophilic cytoplasm and prominent nucleoli. A remarkable degree of nuclear atypia with diffuse hyperchromasia and irregular nuclear contours, and cellular pleomorphism was evident throughout the whole lesion. Architecturally, the tumor featured a mixed pattern of growth, both solid and tubulocystic, occasionally including distinctive papillary areas with hyalinized stromal cores. The clear cells lining the papillae and the cystic spaces showed the characteristic hobnail appearance. A few eosinophilic hyaline globules were observed as well (Fig. 3a–c). Interestingly, sparse foci of endometriosis were present in the fibrous adipose tissue all around. Surgical margins were free of disease.

Immunohistochemical investigation showed positive tumor cell reaction for CK7 (Fig. 3d), EMA, CKAE1/AE3, CD15, CA-125. On the contrary, immunoreaction for Calretinin, WT1, estrogen, and progesterone receptors, cytokeratin 20, CD10, alpha fetoprotein, CDX2, TTF1, and thyroglobulin were all negative. Hence, the morphologic findings and the immunohistochemical results were consistent with a diagnosis of clear cell adenocarcinoma most likely arising in the setting of an abdominal wall endometriosis. Lymph node involvement was documented in 7 out of 14 pelvic lymph nodes, and in 8 out of 11 inguinal lymph nodes.

After 2 months from surgery, the patient was submitted to total body CT scan in order to plan subsequent adjuvant treatment. Despite the absence of disease in the pelvis, CT scan documented the appearance of six neoplastic lesions (from 2.7 to 5.5 cm maximum diameter) in the liver. Treatment with pegylated liposomal doxorubicin (20 mg/m^2^, biweekly administration) was started, and CT scan evaluation after 3 cycles showed the progression of hepatic involvement and appearance of ascites. The death of the patient disease occurred 1 month later.

## Discussion

We have provided an additional patient to the series of 22 cases of endometriosis-associated CCC arising within CS scar, as reported in the literature [4–24]. Only 2 reports were not available from the requested authors.

Despite the rarity of this condition, the number of reported cases has increased over time (see Table 1) likely due to a higher attention focused on this disease, but also to the increased rate of CS and uterine surgeries documented over time. Therefore, more diffuse awareness of this condition and better understanding of its pathogenesis could be important to provide early diagnosis and more effective treatment.

**Table 1:** Clear cell carcinoma arising from endometriosis of the scar caesarean section

	Age	Previous uterine surgeries	Months since symptoms	Size cm	FNA or biopsy	Primary treatment	Pathology	Adjuvant treatment	Relapse	Death
Schnieber Agner-Kolb 1986 [4]	40	1 CS 15 years before	–	–	–	WE, BSO, Hys	Mass: CCC + endometriosis Ovaries and uterus: negative	RT, progestins	–	Yes after 18 months
Hitti, 1996 [5]	46	1 CS 14 years before 1 CS 12 years before	–	6	–	WE, BSO, Hys	CCC + endometriosis	RT	No after 30 months	No after 30 months
Miller 1998 [6]	38	1 hysterotomy 9 years before 1 abortion 3 years before 1 CS 2 years before	8	4	CCC + endometriosis	WE, BSO, Hys omentectomy	Scar: CCC Ovaries, uterus, omentum: negative Margins: close	CIS-based CT RT	No after 60 months^d^	No after 60 months^d^
Park 1999 [7]	56	1 CS 24 years 1 CS 20 years	–	5	–	WE	Mass: CCC + endometriosis	RT	–	–
Ishida 2003 [8]	56	1 CS 24 years 1 CS 20 years	7	10	Endometrial carcinoma	WE, Hys, BSO	Mass: CCC Ovaries and uterus: negative	CIS-based CT	–	Yes after 24 months^b^
Sergent 2006 [9]	45	1 CS 25 years 1 CS 23 years 2 excisions of benign endometriosis nodules at the scar	17	20	–	WE, BSO, later on: endometrial curettage	Mass: CCC + endometriosis Right ovary: benign endometriosis; left ovary and uterus: negative Margins: 1 cm free	Not done	Yes early after surgery^a^	Yes after 6 months
Alberto 2006 [10]	38	1 CS 11 years BSO, Hys for endometriosis	6	6	–	WE	CCC	Carbo/PTX RT	–	–
Razzouk 2007 [11]	46	1 CS 26 years before 1 CS 24 years before 2 excisions of endometriosis nodules at the scar	–	>20	–	GnRh analogue without benefit WE, BSO	Mass: CCC + endometriosis Ovaries: negative LN: 1 positive	Carbo/PTX	Yes during CT	Yes after 6 months^b^
Rust 2008 [12]	42	3 CS Hys 5 years before	24	5	Carcinoma	WE	Mass: CCC + endometriosis Margins: 1 mm free	Not done	–	–
Bats 2008 [13]	38	1 CS 13 years before 1 excision of endometriosic nodule at the scar	–	10	Atypical cells	NACT (carbo/PTX) No benefit WE, BSO, Hys, omentectomy	Mass: CCC + endometriosis Uterus: adenomyosis Other specimens: negative Margins: 2 mm free	Not done	Yes after 4 months^a^	–
Williams 2009 [14]	53	1 CS 17 years before	24	2.5	CCC (excisional biopsy)	WE of the scar, BSO, Hys, omentectomy Inguinal/Pelvic LNctomy	Mass: CCC Uterus, right ovary, omentum: negative, left ovary: teratoma Inguinal LN: 7 positive/11 Pelvic LN: 8 positive/14	Carbo/PTX (4 cycles)	Yes after 3 months^a^	Yes after 11 months^b^
Bourdel 2010 [15]	43	1 CS 20 years before 1 CS 15 years before 1 excision of endometriosic nodule at the scar	9	9	–	WE, partial resection of pubic symphysis, umbilicus, right rectus abdomen, pelvic LN sampling Later on: BSO, Hys	Mass: CCC + endometriosis Pelvic LN: multiple positive Ovaries, uterus: negative	Carbo/PTX (6 cycles) RT	Yes after 6 months^d^	Yes after 22 months^b^
Yan 2011 [16]	41	2 CS 5 years before 2 excisions of benign endometriosic nodule at the scar 1 year and 4 months before	4	9	Not done	Progestins without benefit WE	Mass: CCC	CT	No after 24 months^d^	No after 24 months^d^
Li 2012 [17]	49	1 CS 26 years before	25 years	9	Not done	WE, Hys, BSO	Mass: CCC Uterus, ovaries: negative	Carbo/PTX (6 cycles)	No^c^ after 8 months	No^c^ after 8 months
Mert 2012 [19]	42	Tubal ligation, right ovariectomy	–	15	Tumor cells Mullerian origin	NACT (carbo/PTX) WE, left SO Hys, left Pelvic LNctomy Omentectomy	Mass: CCC + endometriosis Other organs: negative Margins: free	Not done	No after 1 month^a^	No After 1 month^a^
	51	2 CS Hys for myomas	12	6	Excisional biopsy CCC + endometriosis	BSO, Omental biopsy	Negative Margins: free	RT	No after 31 months^b^	No after 31 months^b^
Shalin 2012 [18]	47	1 CS	10	3	CCC	WE, left ovary cystectomy, endometrial biopsy, pelvic LN sampling	Mass: CCC + endometriosis Ovarian cyst: endometriosis Endometrium: negative Pelvic LN: 2 positive/4 Margins: positive	CIS-based CT (6 cycles) RT	Yes after 5 months^b^	No after 7 months^b^
Ijichi 2014 [20]	60	1 CS 37 years before 1 CS 35 years before	48	4	Atypical cells	WE	Mass: CCC + endometriosis Margins: free	Not done	Yes after 8 months^a^	No after 23 months^a^
Aust 2015 [21]	47	1 CS 16 years before vaginal Hys 10 yearsbefore	6	10	–	WE Later on: BSO, pelvic, aortic LNctomy omentectomy	Mass: CCC Ovaries and omentum: negative LN:2 positive/48 Margins: free	Carbo/PTX (6 cycles)	No after 10 months^c^	No after 10 months^c^
Heller 2014 [22]	37	1 CS 1 CS 1 CS 8 years before	96*	18	CCC	WE, left SO, pelvic LNctomy	Mass: CCC Ovary: negative LFN: multiple LN positive	Refused treatment	Yes after 5 months^a^	–
Liu 2014 [23]	39	1 CS 1 excision of endometriosic nodule at the scar	60	6	–	WE, partial cystectomy, BSO, Hys, omentectomy, inguinal,pelvic, aortic LNctomy	Mass: CCC + endometriosis Ovaries, uterus, omentum: negative Bladder: positive Pelvic LN: 18 positive/21 Aortic LN: 6 positive /6 Inguinal LN: 8 positive/8	Carbo/PTX (3 cycles)	YES after 10 months^c^	Yes after 12 months^c^
Sosa-Duràn 2015 [24]	45	1 CS 1 CS 1 CS	6	9	–	WE, margins: 2 cm free	Mass: CCC + endometriosis	Not done	No after 16 months^a^	No after 16 months^a^
Current case	44	1 CS 9 years before	8	22	Endometrial carcinoma	NACT (carbo/PTX) WE, BSO, Hys, inguinal, and pelvic LNctomy	Mass: CCC + endometriosis Ovaries, uterus: negative Pelvic LN: 7 positive/14 Inguinal LN: 8 positive/11 Margins: free	Not done	Yes after 2 months from surgery	Yes after 6 months

*CCC* clear cell carcinoma, *CS* caesarean section, *WE* wide mass excision, *BSO* bilateral salpingo-oophorectomy, *SO* salpingo-oophorectomy, *Hys* hysterectomy, *LNctomy* lymphadenectomy, *LN* lymph node, *Carbo* carboplatin, *CIS* cisplatin, *PTX* paclitaxel, *RT* radiotherapy

*The mass was reported to have come and gone over the last 8 years since the last CS

^a^From surgical resection

^b^From initial diagnosis

^c^Since completion of chemotherapy

^d^Not specified

Even though the criteria for the diagnosis of endometriosis-associated malignancies include also the coexistence of neoplastic endometrial tissue and endometriosis, almost one third of CCC arising within CS scars were not associated with endometriosis foci (Table 1). However, it has to be considered that four patients had referred cyclic pain in the CS scar during menses, or the cyclic increase/decrease of the mass volume, which could be highly suggestive of the presence of endometriosis implants [14, 17, 19, 23]. Moreover, previous history of the excision of benign endometriosis foci at the CS scar was reported in five cases [9, 11, 13, 15, 16]. Therefore, the absence of pathologically assessed endometriosis foci could be interpreted as either a sampling problem or a consequence of the complete replacement of normal tissue due to massive neoplastic proliferation. In this context, careful collection and evaluation of patient history would be important to have a high index of suspicion for endometriosis-associated malignancy.

Indeed, these masses usually reach very large dimensions (median diameter: 9 cm, range 2.5 up to 22 cm) (Table 1) before the diagnosis is made, thus highlighting the difficulties to suspect this condition and obtain an early diagnosis. This is clinically relevant since primary surgical treatment very frequently requires wide surgical excision of the mass together with partial removal of part of the abdominal muscles and reconstructive surgeries with a mesh or even pedicle-skin-muscle flap. Demolitive surgery was also necessary in the 3 cases initially triaged to neo-adjuvant chemotherapy ([13, 18] current case), since only 1 of them achieved partial response after 8 cycles of carboplatin/paclitaxel chemotherapy [18]; in addition, wide excision was necessary in the 2 cases who had undergone excision of endometriosis nodules at the CS scar in the past, and had been unsuccessfully treated with GnRh and progestins, respectively, before being triaged to radical surgery which led to definitive diagnosis of malignancy. Besides wide resection of the mass, other surgical procedures were often carried out including BSO (82.6%), as well as hysterectomy (71.4%), or endometrial biopsy (9.5%, and omentectomy or omental biopsy (30.4%), in order to exclude other primary tumors sites.

Despite the aggressiveness of surgery and the multimodal treatment approach, of 18 cases with available follow up data, 10 experienced relapse of disease, mostly at distant sites, and 8 patients died of disease.

Apart from the long time interval to diagnosis and the wide extension of disease in the abdominal wall, clinical aggressiveness is also sustained by the intrinsic biologic aggressiveness of CCC, which differs from other endometrial cancer histotypes. In this context, proper diagnosis should take advantage of immunohistochemical panels with several markers in order to exclude serous histotype as well as mesothelial tumors such as malignant mesothelioma and papillary mesothelioma which are usually positive for Calretinin, WT1, and keratin 5/6, contrary to what is observed in CCC. In addition, WT1 is the most important immunohistochemical marker to distinguish serous carcinoma from CCC.

It has to be acknowledged that of 9 cases undergoing lymphadenectomy or sampling, 8 showed diffuse metastatic involvement of inguinal, and/or pelvic, and/or aortic lymph nodes ([11, 14, 15, 19, 22, 23] current case).

On the other hand, some cases were reported to experience relatively longer disease-free and overall survival (≥30 months) [5, 6, 16, 18]; while recognizing that a more thorough molecular characterization could hopefully help define prognosis of this very rare condition, it has to be acknowledged that cases with better outcome presented with masses ranging between 4 and 9 cm, thus suggesting that a prompt recognition and treatment could make the difference.

Obviously, also prevention of endometriosis implantation at time of CS is of utmost importance: as recently emphasized, the uterus should not be exteriorized, exposure of endometrial mucosa during uterus suturing should be limited, and peritonization may be advised, although there is no definitive data about these issues [15].

## Conclusions

Attention should be focused on careful evaluation of patient history in terms of pelvic surgery, and symptoms suggestive of endometriosis such as repeated occurrence of endometriosis nodules at CS scar, or cyclic pain, or volume changes of the nodules.

## Abbreviations

AUC: Area under curve
BSO: Bilateral salpingo-oophorectomy
CB: Carboplatin
CCC: Clear cell carcinoma
CIS: Cisplatin
CS: Caesarean section
FNA: Fine needle aspiration
HYS: Hysterectomy
LN: Lymph node
LNCTOMY: Lymphadenectomy
MRI: Magnetic resonance imaging
PET/CT: Positron emission tomography/computed tomography
PTX: Paclitaxel
RT: Radiotherapy
WE: Wide mass excision
